# Music Use for Mood Regulation: Self-Awareness and Conscious Listening Choices in Young People With Tendencies to Depression

**DOI:** 10.3389/fpsyg.2019.01199

**Published:** 2019-05-24

**Authors:** Joanna Stewart, Sandra Garrido, Cherry Hense, Katrina McFerran

**Affiliations:** ^1^MARCS Institute for Brain, Behaviour and Development, Western Sydney University, Penrith, NSW, Australia; ^2^Department of Music Therapy, Faculty of Fine Arts and Music, The University of Melbourne, Melbourne, VIC, Australia

**Keywords:** young people, music, mood regulation, depression, self-awareness

## Abstract

The current study explored the circumstances in which seven young people with a tendency to depression chose different styles of music to listen to, and their level of awareness of the impact of their music listening habits on mood and wellbeing. A model of various pathways through music use was developed that may explain why music listening intentions in young people do not always align with their wellbeing outcomes. We suggest that the relationship between intentions and outcomes are mediated by differing levels of self-awareness and insight into the mood regulation processes occurring during music listening.

## Introduction

Depression is responsible for the deaths of many people globally each year, with suicide being the leading cause of death around the world in 15–29 year-olds (WHO, 2017). Many more young people experience depression at highly debilitating levels, around 8% in Australia meeting the DSM criteria for Major Depressive Disorder (MDD) (Lawrence et al., 2015), and 13% in the United States (National Institute of Mental Health, 2017). The early onset of depression is a critical factor in terms of projected quality of life (Sullivan et al., 2012), and if left untreated, depression can become a lifelong disability (Meade and Dowsell, 2016). Depression also has an impact on the social and intellectual development of young people as well as reducing engagement with education at a crucial developmental stage. It is therefore imperative to address depression in young people before its impact on their lives increases.

### Depression and Media Use

Access to online media has increased exponentially with the onset of digitisation and technological advancement (Brown and Bobkowski, 2011). Research has demonstrated that young people are even more likely to turn to media when they are in a negative mood (Dillman Carpentier et al., 2008). In fact, withdrawal from socialization and normal daily activity has been identified as a behavior consistent with clinical depression and this often involves an increase in general media use (O’Keeffe and Clarke-Pearson, 2011). This increased engagement with media includes music listening, with emotional dependency on music also tending to increase during periods of depression (McFerran, 2016).

However, research has demonstrated that this increased reliance on music during episodes of psychological distress does not always have positive mental health outcomes for the young people involved. For example, Garrido and Schubert (2015a,b) have demonstrated that people with a ruminative coping style, which is highly predictive of clinical depression, tend to be attracted to music that can intensify symptoms of depression. Similarly, in a study by McFerran et al. (2015) the authors discovered that having high levels of distress while listening to music was associated with more intense, negative moods afterward. Other studies confirm the fact that people with depression are not always able to effectively select music that helps them to feel better (Wilhelm et al., 2013; Hense et al., 2014). They may also use music as part of generally unhealthy coping strategies such as emotion-focused coping (Miranda et al., 2012), rumination (Garrido and Schubert, 2013), or social withdrawal (McFerran and Saarikallio, 2014).

### Self-Awareness and Depression

Self-awareness can be described as clear awareness of one’s own feelings, emotions, and behaviors (Blakemore and Frith, 2003). Such awareness is generally regarded as an adaptive function that can result in identification of aspects of the self that would benefit from modification. Experiencing feelings of sadness can often provide the motivation for self-scrutiny and behavioral modification, even increasing detail oriented thinking and realistic thinking that is useful for problem solving behaviors (Keedwell, 2008). However, in depression, the adaptive function of sadness tends to malfunction, with depression being associated with increased pessimism and reduced motivation to engage in problem solving (Bianco et al., 2013).

In general, individuals differ as to their levels of cognitive insight, or their capacity to understand their own thoughts, behaviors and affective states (Riggs et al., 2012). However, research has demonstrated that low emotional awareness is highly predictive of depression and anxiety in young people (Kranzler et al., 2016). Emotional awareness, or the ability to identify emotional experiences, can be a protective factor against psychopathology by allowing an individual to recognize the need to activate appropriate emotion regulation strategies (Barrett et al., 2001). On the other hand, young people with low emotional awareness tend to have reduced access to effective strategies for coping with negative affect and interpersonal difficulties (Flynn and Rudolph, 2014).

This lack of awareness may also play into music listening choices in young people with depression. This is implied by one study conducted by Garrido and Schubert (2015b) in which participants with high levels of rumination reported having benefited from listening to sad music while at the same time reporting an increase in depressive symptoms. Similarly, in a study on listening to nostalgic music, Garrido (2018) found that implicit mood measures (in which participants are unaware that their mood is being assessed) indicated a much higher level of negative mood responses after listening to nostalgic music than participants reported in response to direct questioning. The issue of the discrepancy between perceived and real mood changes was also discussed by McFerran et al. (2016) in a systematic review of 33 articles about music and mental health. Their review revealed that while direct questioning usually suggested positive mood effects from listening to music, non-direct mood indicators suggested results were not always so positive. At times this appeared to be because researchers worded questions in such a way as to suggest positive effects. In other cases study participants demonstrated a tendency to construe music listening positively regardless of its effect on their mood. This reveals issues both with demand characteristics in study design as well as a degree of positive bias in participants.

In exploring the concept of awareness further, McFerran and Saarikallio (2014) identified three different response styles with regards to music choices. They found that some people can recognize that the music they listen to is not beneficial to their mood and then be proactive in changing their listening habits. The second response style is when a person can be made aware of deteriorations in their mood by others and change their habits. This has been shown to be possible, for example, with young people who are seeking help for depression and who work with a music therapist to identify more helpful ways of listening to their preferred music (McFerran et al., 2018). The third response style is when a person may either recognize or be made aware of the negative impact but is not inclined to modify their listening behaviors. Alternatively, if an individual’s mental health is very poor, they may not be able to focus on therapeutic interventions that demand high cognitive function such as this level of meta-reflection on intentional music listening (Hense et al., 2018). Thus, it appears that there is a need to develop nuanced strategies for increasing awareness of the effect that music listening can have on young people’s mood and wellbeing. Given the central role that music plays in the lives of young people, increasing such awareness has the potential for positive benefits through increased understanding of adaptive and maladaptive behaviors more generally. There is a need to further understand how young people are enabled to increase their awareness about the effects of music on their wellbeing.

The current study uses a grounded theory approach to explore the following research question through interviews with seven young people: To what degree are young people with symptoms of depression aware of the effect their music-listening has on mood and wellbeing, and how do they reach a state of awareness?

## Methods

The research question lends itself to an inductive approach in which a topic is explored with no prior hypothesis. Grounded theory is one qualitative method that is often used to investigate the ways in which various conditions interact with an individual’s experience of a given phenomenon, with an emphasis on analyzing people’s actions and integrating what they do as well as what they say (Charmaz and Bryand, 2007) This method enables theoretical notions to be extrapolated from qualitative data, rather than generating a rich description (Dey, 2007). In grounded theory, the researchers endeavor to approach the data without being influenced by *a priori* knowledge. Through processes of coding, constant comparison and abstraction of concepts from data categories, a conceptual hypothesis or theory can be developed (Chun Tie et al., 2019).

### Participants

Participants were recruited from among people who had taken part in an online survey and had indicated their interest in being involved in further research (Garrido et al., 2017). Initially, 615 people participated in the survey and were asked to complete the Depression and Anxiety Stress Scale (DASS; Henry and Crawford, 2005) and the Rumination-Reflection Questionnaire (RRQ; Trapnell, 1997). Purposive sampling was used, and potential participants with DASS scores above 15 and rumination scores above four were approached, as these are indicative of severe symptoms of depression and ongoing ruminative coping styles. This generated a list of 27 potential participants, 4 of whom were not approached because they had indicated being negatively affected by participating in the survey. The participants on the inclusion list were contacted by email, with 7 young people aged from 19 to 28 years (mostly female) responding and being interviewed. Participant demographics are included in Table 1. As the previous study had been conducted online the participants lived in a number of different countries.

**Table 1:** Demographic information of interview participants.

Participant #	Age	Sex	Previous treatment for depression	Country
1	28	Female	No	Australia
2	19	Female	Yes	United States
3	24	Female	No	United States
4	22	Female	No	Brazil
5	19	Female	No	United Kingdom
6	22	Female	Yes	Canada
7	23	Male	No	United Kingdom

### Materials and Procedure

Ethics approval was granted by the Human Research Committee (#1443393.1) at the University of Melbourne. Potential participants were contacted via email and if they responded with interest were sent a Plain Language Statement and Consent form. Written informed consent was obtained from all participants prior to conduct of the interviews. Due to the diverse locations of participants, all but one interview was conducted via the video call function on Skype using Version 7.39.0.102. Audio of the interviews was recorded using MP3 Skype Recorder 4.32 Free Edition. Since all participants were fluent English speakers interviews were conducted in English and took approximately 45 min to 1 h. Participants were offered a $15 iTunes voucher for participating in the study.

Interviews were conducted by the first two authors and memos were created after each interview to record the impressions of the interviewers about participants’ demeanor, body language, tone of voice and time taken to respond as well as the interviewers’ reflections on how their own personal biases may have influenced their conduct of the interview (Finlay, 2002). Discussions between the first two authors took place after each interview to determine the direction of the subsequent interview. An interview guide was used, however, interviews proceeded freely based on participant responses (see Appendix). In general, the interviewer sought to prompt participants to discuss their use of music to regulate their moods, and in particular negative moods. In order to determine the level of awareness and responsiveness to the idea that music does not always have positive effects on mood, and to avoid the positive bias that has limited some previous studies, the interviewers deliberately introduced the topic of negative effects toward the end of the interview if participants had not raised the issue themselves.

### Analysis

Interviews were transcribed verbatim. A preliminary analysis of each interview was conducted using open coding in accordance with Strauss and Corbin (2008). The initial *in vivo* codes related primarily to the ways the young people described using music to regulate mood and included codes such as “for comfort,” “music is used as a distraction” and “to keep fighting.” This initial coding allowed the first author to immerse herself in the data and revealed new questions for consideration in subsequent interviews to enable deeper exploration of new issues. For example, themes of anxiety began to emerge as common around the fourth interview, and so additional questions in relation to this were added to the interview guide for subsequent interviews.

Once open coding of the interviews had been completed a second wave of analysis was conducted independently by two pairs of researchers (the first two and last two authors) who each identified themes and central categories that connected the various ideas that were emerging from the data. Axial coding as described by Charmaz (2006) was then used to discern possible relationships between the various categories and codes, with constant return to the data to better understand whether similar coding was representative of shared ideas. The codes were examined to assess the motivations participants gave for adopting particular mood regulation strategies with music and the factors influencing the relative success of these strategies. This informed the generation of sub-categories within the larger concepts that had been identified across participants. The properties and dimensions of the categories and sub-categories were then delineated with a focus on the central code of awareness and, where present, how this awareness was described as developing. Memos were used as a way of noting impressions from the data, with statements such as “It seems like…” The analysts would then return to the raw data to see whether these impressions could be sustained by what had actually been said. This process allowed for a constant attention to the possibility of the researchers’ pre-assumptions influencing interpretation of the data, rather than allowing details about the phenomenon to emerge from the participants’ experiences, which was the focus of this study. The two research teams then compared their analyses and used selective coding to integrate and further refine the central category, it’s properties and dimensions and to develop a theoretical proposition.

### Reflexivity

Since researchers who have worked in a particular field for some time may already be familiar with previous findings in this area and be somewhat influenced by their knowledge, reflexivity was considered an important part of the research process (Finlay, 2002). Qualitative analysis is an inherently subjective process and two of the authors (SG and KM) have undertaken a number of studies of this topic previously. It was therefore critical to ensure that analysis was not used to simply confirm our existing beliefs (confirmation bias). Undertaking two separate analyses was our primary strategy for testing our ability to focus on what was being said by the seven participants and we frequently returned to the data to scrutinize our emerging ideas and test how closely it matched the raw data. This allowed us to rigorously debate and refine our presentation of the findings. Throughout the interviews and analysis process, the researchers also engaged in personal and shared reflections and debate about potential meanings inherent in the data.

## Findings

### Strategies for Music Choice

**FIGURE 1:**
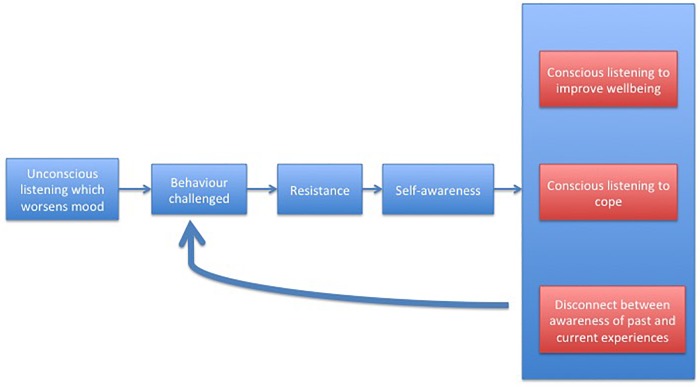
Phases of awareness and their influence on music listening strategies.

Our analysis suggested that the strategies participants described for using music to manage negative moods fell into two broad categories: (i) selecting music that differed from the negative mood in an effort to shift a negative mood, and (ii) selecting music that mirrored the negative mood in an effort to cope with negative feelings. These strategies are depicted in Figure 1 following the model of Biasutti (2013, 2018). Both strategies appeared to have negative outcomes at times and positive outcomes at other times.

#### Music That Differed From Current Mood

Many (5 of 7) of the participants described listening to music that differed from the mood they were experiencing in order to try to alter a negative mood. For example, Participant 3 described listening to classical music when angry to help her calm down: “If I’m listening to classical music because I’m trying to calm myself down, it’s soothing. It helps me relax. It’s more like trying to relax, that I’m feeling the music and trying to absorb every note.” Similarly, several participants identified using calming music to reduce feelings of anxiety. Participant 5 stated “I have anxiety issues so I find it quite a nice way to settle myself,” while Participant 7 described using music to “get out of my head” when feeling anxious. Participant 2 also reported sometimes using music to “block out things that are bothering me.” Participant 5 also described successfully listening to upbeat music when feeling down. She stated: “If I’m feeling depressed I tend to put on happy music like cheesy pop and things to try and cheer myself up almost. Something with a fast tempo to kind of boost my mood.” These strategies were considered to represent conscious processes adopted by participants to change their mood. In contrast, Participant 4 reported that listening to music that didn’t match her negative mood gave her “the impression that everyone else is having fun except for me.”

#### Music That Mirrored Current Mood

The other prevalent theme we perceived across most (6 of 7) participants was the use of music to mirror mood in an attempt to cope with feelings of sadness and depression. While the term ‘cope’ can cover a wide range of strategies for dealing with undesirable situations and affective states including problem-solving and attempting to change one’s mood, in the context of this data the term is used to describe strategies designed to mitigate or lessen the intensity or unpleasantness of an undesirable mood without actually shifting it’s valence. Different participants described selecting music that mirrored their current mood in relation to a diverse range of intentions or aims, and outcomes. However, our interpretation was that these strategies often appeared to be designed to help participants cope with affective states, rather than to change them.

Some participants expressed the desire to be “comforted” when listening to music, gaining understanding that “I’m not the only one going through problems” (Participant 3). This strategy provided a feeling of “validation” of the experience for Participant 4, and Participant 6 described something similar saying, “I’m tired and I’m still sad but it’s less heavy and it’s like someone understands.” Participant 3 also reported that sometimes it was just a matter of changing the degree of sadness being felt, describing how in the past she found it useful to listen to music that was “at a level that is just a little bit above what I’m feeling, to maybe bring me up a little bit but not so much that it would bother me.” In this case, even a small improvement in mood was perceived as a positive change.

Others listened to mood-matching music with the express aim of intensifying their negative emotions. Several participants reported listening to slow, acoustic, classical pieces to reflect and emphasize a sad or low mood state. For example, Participant 1 described deliberately choosing songs that conveyed “extreme manifestations” of the sad mood she was in. Two participants described their motivation as being to “drown in” (Participant 3) or “wallow in” the negative emotions (Participant 4). Participant 3 described how this had a positive effect, and how it “gets me to the highest point and then I come down,” suggesting that she experienced some relief once the more intense emotions diminished. For Participant 1, the effect was less clear and she reported listening to music with suicidal lyrics when depressed, stating that this “probably just intensified the emotion, which [may] or [may not] be beneficial.” Participant 4 similarly reported that this could leave her not even feeling “motivated enough to change the music.”

In contrast to the others, Participant 2 reported that she preferred to avoid music that could make her feel more depressed. This did not necessarily entail listening to music that was upbeat and happy, which she tended to listen to when having a “better day,” but she said: “If I’m not feeling so good I’ll listen to a classical piece, something slow.” She also made the following statement.

“I try not to listen to depressing music if I’m already feeling down because it’s not going to do anything to help really. Sometimes I kind of need it to know that other people feel the same way, but a lot of time its just going to make me feel worse and so then I don’t want to do that… Usually I listen to more positive things.”

### Factors Influencing the Outcomes

Our analysis of the properties and dimensions of the strategies presenting in the data revealed that both positive and negative effects were experienced from both strategies for managing moods with music. This was determined during axial coding, where we examined the circumstances surrounding these strategies in order to determine some of the factors that contributed to a positive or negative outcome. We identified three properties: (i) the messages conveyed by the lyrics, (ii) the frequency and duration of listening to certain music, (iii) the nature and intensity of the prior affective state of the listener.

#### The Messages Conveyed by the Lyrics

Some participants described particularly being attracted to music with lyrics that have special meaning for them when feeling down. When Participant 1 described the kind of music she was drawn to in a depressed mood, she said “It’s both the music and the lyrics as well, and I think that what the singer’s expressing is a sort of frustration and I’ll think, ‘Oh yes, that’s exactly what we feel here’.” Participant 6 described how music with lyrics was especially important to her when she was feeling sad: “When I’m really, really sad that’s the only time I’ll listen to music where I care more about the lyrics.”

However, the outcomes weren’t necessarily positive for participants when they listened to music with lyrics that closely related to how they felt. Participant 1 stated: “I started thinking about the lyrics and stuff and it’s not pleasant stuff and I began to think, well maybe it’s listening to this stuff which is really contributing to my being in a very low mood.” Participant 6 similarly said: “I was just getting really perturbed because I was listening to the lyrics too much and I could relate and then I could go and watch TV or something but I kept thinking about the song.”

More positive effects were noted when listening to songs that were considered “emotional but” also inspired “some optimism,” or had an “uplifting message” (Participant 6). Participant 2 stated that “If the music has a positive outlook on life, I’m likely to kind of adopt that somewhat.” Thus, several participants demonstrated how the differing messages in music, even music that mirrored their mood, could have differing effects on their mood.

#### Frequency and Duration of Music Listening

Participant 1 explained how she had experienced a phase in her life when she was listening intensely to music with very negative, suicidal lyrics. She made the following statement.

“I was listening for hours a day…to be hearing people talking about drug problems, how much they don’t like themselves, that they were locked up in the mental hospital etc. On a daily basis that probably isn’t the world’s best for mental health.”

Participant 2 similarly described that the amount of time she spent listening to sad music needed to be limited. “Sometimes I kind of need it to know that other people feel the same way… But there does come a point when you are feeling bad enough and then that would make you feel worse and it’s something you have to stay away from.” Participant 6 also mentioned a time when she had been listening to songs about suicide “quite a few times…too much” with negative results. Thus, there was a recognition that intense or frequent listening to music that reflected negative thinking was likely to have a more negative impact on wellbeing.

#### Nature and Intensity of the Prior Affective State

Being in a low negative mood state was frequently mentioned as a factor that would result in music listening having a negative or neutral effect on mood. For example, Participant 4 said: “If I am having a really bad day then nothing I do will really change that.” She also stated that, “When I’m in a more neutral mood I can change my emotions according to what I’m listening to but when I’m really sad nothing helps.” Similarly, Participant 7 commented that it’s the strength of his mood that influences how easy it is to modify with music: “Some moods are harder to shake.”

A number of participants described using music to distract from, or mask unpleasant emotional states. For example, Participant 6 described herself as having “an anxiety disorder” and being able to use music to “distract” herself or “calm” herself down. Although this strategy was sometimes described as helpful in alleviating the intensity of emotional experiences, some participants also acknowledged the temporary nature of this solution. For example, Participant 7 stated that music could not remove anxious feelings altogether, but that it would just “temporarily mask the depression” and then he would “be back to square one” when the music finished. Similarly, Participant 6 stated that “on the spot it’s useful because I’m just thinking about the music, I’m forcing myself not to think about what’s making me anxious but when I stop everything comes back.” Thus, some participants identified that ‘covering up’ emotions were a short-term and somewhat limited solution.

Strategies also appeared to differ depending on the individual’s mood. Participant 3 said that when she was feeling sad she usually chose music that matched her mood because she found it “comforting” and “reassuring.” However, when angry she would listen to music that she hoped would change her mood.

### Awareness

In examining some of the factors that influenced the outcome of music listening for people with symptoms of depression, it seemed possible that a key factor was the level of awareness and consciousness with which individuals selected music. Selective coding allowed us to explore the data in order to further test this theory. In order to overcome previous study limitations which have demonstrated that individuals do not always directly report negative effects of listening to music, coding strategies here looked not only at clear statements relating to awareness, but at other indications such as inconsistent responses or signs of ambiguity or confusion. Upon direct request, most participants were able to list a song or a type of music that had previously caused a deterioration in mood for them. For example, Participant 4 reported that listening to “emo” music had previously had a negative effect on her mood. Participant 1 similarly described listening to Elliott Smith and realizing that her mood was “continually low.”

It was evident that for several participants their insight into the potential for music to have negative effects was something they had gained over time, usually after some negative experiences. For example, Participant 5 stated:

“It’s something I’ve developed over time. It’s like a mechanism I’ve developed as I got more used to having mental health problems…I used to listen to a lot of punk rock stuff and all that kind of emo stuff nonsense and it just used to make me much more worked up because it’s so intense that it does not help. But it took me quite a long time to realize what was happening.”

Awareness was obtained in several ways. Some participants were made more aware by the comments of friends and family. Participant 5 made the following statement.

“My family members were like, ‘This is too intense. Why are you listening to this? You are obviously struggling.’ There is a history of mental illness in my family so they are quite good at knowing how to deal with it. And I was like, ‘No it is helping’… until I heard a few people my own age say that it’s not working.

She similarly said: “Some of my friends had some of the same issues and they said how they listened to some music that made them feel worse and I was like “oh maybe they have figured out what’s wrong with me too” and I sort of realized that.”

Of note in Participant 5’s comments is the fact that she had at first believed that the music was helping her, but she ultimately identified with the experience of friends who noted that it was not always as helpful as it could be.

For Participant 6 it was therapy that had helped her to become more aware of her listening habits. She made the following statement:

“When I was younger I did have a tendency to just listen to songs more, especially if they made me sad and they emphasized my mood. Now, I would take a break from them and listen to something different because I’m trying, well not trying to control my mood but it was one of the things I discussed with my therapist. I was not trying not to hurt myself more, but trying to not feel worse about things.”

For Participant 1, the realization appears to have come to her personally without prompting from other people. “I began to think, ‘Well maybe it’s listening to this stuff which is really contributing to my being in a very low mood, so I had to stop then.” She reported realizing that listening to particular music with suicidal lyrics had “brought [her] into a more negative mood.”

However, there was some evidence that despite a recognition of the negative effects of past experiences, this did not always translate into an awareness of the potential impact of current listening behaviors. Several participants seemed to lack clarity in their own mind about whether particular listening was useful or not. This was evidenced by some inconsistent statements within the interviews. For example, when Participant 4 was asked what she listens to when she is in a low mood she said: “emo music.” However, when asked to describe a situation where music had made her feel worse she described how in high school emo music had made her “feel worse.” When asked again whether she would do the same thing now, she said: “Probably not.” In this case, the participant recognized some negative effects in the past but still reported the same listening choices. However, asking the participant to reflect on the past negative experience caused her to change her answer about current listening choices. Whether or not this reflected an actual change in opinion is unclear.

Participant 6 was similarly somewhat ambiguous about whether her listening choices made her feel worse. As cited above, this participant reported discussing with her therapist about avoiding music that made her feel “worse about things.” She seemed to have some useful strategies for regulating her mood such as listening to music that is “sad” but that gives her “some optimism,” or music that gave her some relief in that after listening she was “still sad but less heavy.” However, when speaking about a previous experience she made the following statement.

“I don’t know if it made me feel worse…but there was this one song about suicide that I remember listening to…the song itself kind of hit close to home at that moment… I was just getting really perturbed because I was listening to the lyrics too much.”

When asked if she would listen to the same song again she said “sometimes I do,” but preferred not to listen to it when in a good mood because it “brings back memories.” Thus, while this participant appeared to have some awareness of her complex responses to certain music in the past, she sometimes seemed to revert to unhealthy patterns in periods of depression. Of note with this participant was a recognition that the message in the lyrics – whether suicidal or optimistic – was an important factor even when the music was “sad” overall, with the former tending to make her feel worse, while the latter helped her to feel “like someone understands.”

Similarly, Participant 1 gave some conflicting statements in reference to her preference for listening to Elliott Smith and his music with suicidal themes when feeling depressed. At first when describing her response to this music she stated, “I just kind of think, ‘Oh God, this is just so sad and so depressing.’ I kind of sit there and think ‘oh woe is me’.” She also reported having had to stop listening to this music at one stage because she had been getting too depressed, and stated that the music “probably just intensified the emotion.” However, when the interviewer introduced the question of whether some other listening choices might be more helpful, the participant was quick to justify her preferences stating, “If I listen to depressing music when I’m depressed it does have some benefits.” Music was intensely important to this participant. She reported listening to music much of each day and spending a lot of time reading about musicians and their lives. The type of music she listened to appeared to be closely connected to her sense of identity, and her attraction to music that was meaningful and musically “masterful,” something which she associated with singer-songwriters such as Elliott Smith. Thus, for this participant there appeared to be some resistance to the idea that such music choices could have a negative influence on mental health despite a recognition of past negative experiences.

Participant 2’s approach contrasted with the other participants. She described herself as having been better in recent years, but even when going through a period of depression, her music listening choices did not necessarily have a negative outcome. She described using music as a temporary thing to escape from her difficulties. “I remember there were times when I would find a 12 min piece and just put my headphones on, turn it up, probably too loud, and then sit there and enjoy it for 12 min and that was 12 min that I didn’t have to deal with everything else.” This demonstrated some intentionality of music use, but not necessarily an ability to sustain the wellbeing benefits with music. Participant 4 similarly described her approach to music listening when depressed as “a way of soothing my emotions rather than solving them.”

Thus the participants in general described unconscious listening that worsened their mood as a past way of using music when they had little to no insight about their emerging mental health problems. This suggests that when participants were not aware of how poor their mental health was becoming, they used music in ways that contributed to their deterioration and stopped once something challenged them – the realization of depression, comments by family or friends, or therapy. However, the same listening behaviors sometimes seemed to continue or re-occur in current circumstances especially when there was a deterioration in wellbeing.

### Pathways to More Conscious Music Use

**FIGURE 2:**
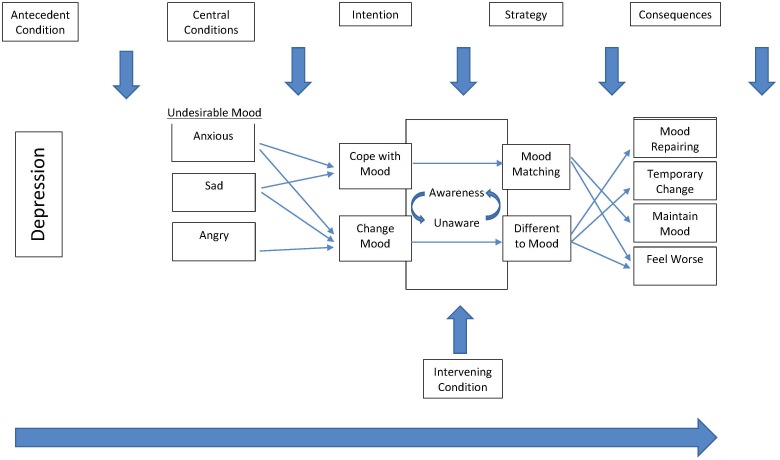
Model of the pathways young people with depression take in using music to deal with negative affective states.

The emphasis placed by grounded theory analysts on actions and interactions encourages modeling of diverse pathways through a phenomenon (Strauss, 1987; Morse et al., 2016). For this study, the findings suggested a model of the pathways young people with symptoms of depression take through music use that reflect differing strategies for dealing with undesirable moods (see Figure 2). The model demonstrates how listeners may have the intention to either cope with or to change an undesirable mood, but outcomes vary depending on the strategy used, which in turn is influenced by the individuals’ fluctuating level of awareness. In this model, individuals have the antecedent condition of depression and are influenced in their music listening selections by the central condition, which is the state of being in an undesirable mood. They then exercise their intention to either cope with their mood or change their mood through strategies that involve selecting either mood matching music or music that is different to their mood. These selections are influenced by their own changing levels of awareness which in turn, are influenced by intervening conditions such as negative experiences or discussions with family, friends, or a therapist. These differing strategies can have varying outcomes, with mood matching music generally leading to either maintenance of mood or feeling worse, and listening to music that is different to the initial undesirable mood generally resulting in mood repair or a temporary change to mood.

## Discussion

This study focused on exploring the degree to which young people with symptoms of depression are aware of the effect their music listening choices have on mood and wellbeing, and how they reach that state of awareness. Our findings demonstrated that most young people in our study reported past behaviors reflecting limited awareness and unconscious motivations, often with undesirable outcomes. However, intervening conditions including insights gained from friends, family, a therapist or through self-reflection, resulted in some increased awareness. Previous research has demonstrated that increased awareness of the effect of music listening choices can be deliberately influenced, such as through use of the Healthy-Unhealthy Music Scale as an awareness raising tool (Saarikallio et al., 2015; McFerran et al., 2018).

However, in the current study, some young people demonstrated an initial resistance to increased awareness, or a reversion to previous unhelpful patterns of music listening even after reaching a level of awareness, particularly during depressive episodes. Thus, the pathways young people took through music listening and awareness of the effects of their music listening choices were not always linear. Skill building appeared to be a gradual process of discovery by continual cycling through varying intentions, strategies and outcomes, with new levels of consciousness being reached as new events and experiences challenged old behaviors.

Of note in the current study was that some participants described being more able to use music to change states such as anxiety or anger even when these were quite severe, but were less inclined to use this strategy when feeling depressed. When seeking to change an anxious mood, for example, participants reported listening to calming music – music that did not match their current mood. In contrast, when dealing with depression, many of the participants reported using music that maintained their current mood in order to feel validated and to have their feelings acknowledged.

While the intention of participants was to use music to help them cope with their depressed mood, this sometimes resulted in intensifying the state. It may be that the different interactional strategies used in each case contributed to the differing outcomes since research suggests that listening to mood shifting music is often more effective than listening to sad music when feeling depressed (Garrido and Schubert, 2015b). Alternately, it may be that anxiety is more amenable to influence by music listening. In a systematic review of studies relating to music and people with dementia, for example, it was found that music can reliably reduce agitation in patients, while the effects of music on symptoms of depression are less consistent (Garrido et al., 2018). Anxiety is often exacerbated by a fear of the symptoms of anxiety themselves (Dugas et al., 2012), but calming music can reduce physiological symptoms of anxiety thereby inducing a relaxation response (Hamel, 2001). On the other hand, depression is often closely related to thought patterns which may be less likely to be altered when listening to music, particularly if the music echoes the existing negative thoughts.

It may be this relationship between thought patterns and depression that can help explain why the benefits of listening to distracting music was sometimes time-limited, ceasing as soon as the song was over. This has further been noted in Cheong-Clinch’s study of adolescents with mental illness (Cheong-Clinch and McFerran, 2016), where music was found to mediate mood momentarily, but it was more difficult for young people to achieve sustained benefits. As reflected in our findings, the content of the lyrics often had an influence on whether positive benefits were achieved. When participants listened to music that mirrored their current circumstances this appeared to have less desirable mood outcomes, possibly because listening to such music is akin to ruminating. In contrast, outcomes were more positive for participants in the current study when they listened to music with optimistic messages. Previous research has similarly demonstrated that the thoughts triggered by music have a greater impact on mood outcomes than features of the music itself (Garrido et al., 2016). Thus, listening to music that is distracting as opposed to music that alters mood via shifting thought patterns, may be only of temporary benefit.

Nevertheless it is important not to ignore momentary benefits for people struggling with depression. Some theorists suggest that the cumulative benefits of positive moments can serve as protective factors that eventually lead to improved wellbeing (Rutter, 2012). More pragmatically, young people who are struggling with suicidal thoughts appreciate even small periods of escape (Cheong-Clinch and McFerran, 2016). Such brief distractions are helpful in that they reduce time spent ruminating and can reduce the incidence of self-harm and suicide attempts (Polanco-Roman et al., 2015). Furthermore, studies in music therapy have indicated that selecting music that matches one’s mood as the beginning point of a process that gradually shifts toward more positive music – a strategy known as the iso-principle – can produce a more enduring repair of mood (Davis et al., 2008). The participants in the current study did report experiencing some lessening of the intensity of their negative moods after listening to mood-matching music. It is possible that for some, the reduced intensity of their negative moods was the beginning of a process of recovery. However, the data in this study did not reveal this clearly, and it is likely that the long-term outcomes of this process differ from individual to individual particularly in situations where the person has a high level of unawareness about the thinking patterns and emotions being triggered by the music.

### Clinical Implications

While some participants in this study described reaching awareness of their strategies for music use on their own, external input such as from friends, family or a therapist was also described and has been categorized as intervening conditions in Figure 2. Although this may suggest that telling young people to be more careful about their music listening could be beneficial, a broader cultural context is also at play. Young people report feeling resentful of the judgments made about their music choices, and one function of music is often described as being to assert an independent identity, beyond parental authority (Laiho, 2004). In a previous study, we were able to encourage young people seeking support for depression to contemplate their music listening habits, but this occurred within a respectful conversation that involved both validating music preferences as well as dialoguing about consequences (McFerran et al., 2018).

It is also common for caring adults to mistake the mechanism of action in this scenario and to blame the qualities of the music itself, rather than focusing on how music choices reflect mental health. This has historically been a point of contention between fans of heavier genres, such as Rock and Rap, and correlations are frequently found with antisocial behaviors (Lozon and Bensimon, 2014). Nevertheless, a causal relationship between particular music genres and mental illness or problem behaviors has never been established (North and Hargreaves, 2006). Rather, complex interactions between an array of personal and social mechanisms underlie our emotional reactions to music (Juslin et al., 2015). Interventions that focus on self-reflection and raising awareness of the interaction between thoughts and feelings triggered by our music listening choices are likely to be more successful than those targeting particular music genres or styles.

## Conclusion

There is ample evidence to demonstrate that people use music to improve their mood on a daily basis, both in everyday life (DeNora, 2000; Saarikallio, 2007; McFerran et al., 2015; Papinczak et al., 2015) and in music therapy (Maratos et al., 2009; Cheong-Clinch, 2013; Bibb and Skwews McFerran, 2018). There is also an emerging body of research which seeks to qualify these findings, since it is clear that music is not a magic pill that can immediately resolve a negative mood and nor is it always helpful. This research contributes to this second discourse, highlighting how individual’s uses of music can result in various outcomes depending on a range of factors. Individuals can use music listening to improve, maintain or intensify a mood, and may do any of these things at various times. Although it appears that people with depression are most likely to use music to intensify a negative mood, they are also the least aware of this tendency. This is further complicated by the finding that an individual can become aware of unhelpful listening habits, but can lose that awareness when in a depressive state and revert to intensifying strategies.

The current study is limited by the fact that the sample was primarily female. This gender imbalance is not unusual in studies relating to mental health (see for e.g., Lindner et al., 2016), and is likely a reflection of the higher rates of depression among females (Freeman and Freeman, 2013). Nevertheless, future studies could benefit from recruitment of a more balanced sample so as to explore gender differences in strategy selection and outcomes of music use. Future research should also consider the influence of cultural context. The current study included at least one participant from a non-English speaking background. While music tastes among young people are becoming increasingly globalized (Cicchelli and Octobre, 2017), culture nevertheless has an impact not only on music selections, but on the way individuals value particular emotional experiences (Oishi et al., 2007).

Using music to influence mood is likely to continue to be a popular strategy for many people, both in their everyday life and through music therapy or other therapeutic contexts. Therefore, our ability to predict when this is likely to be more or less helpful and to develop strategies for supporting people during the most difficult moods is critical. However, the nuances of the pathways through music listening and toward an improved mood are complex and need to be individually identified and negotiated. The findings from this research indicate that promoting awareness of the power of music to enhance any mood is helpful, but that we should be prepared for circuitous pathways and open to change in all directions when people engage with their preferred music.

## Ethics Statement

The study was approved by the Human Ethics Committee of the University of Melbourne. Written consent was provided by all participants.

## Author Contributions

SG and KM developed the initial project design. JS and SG undertook the data collection. All authors contributed substantially to data analysis, write up, and development of the conceptual model.

## Conflict of Interest Statement

The authors declare that the research was conducted in the absence of any commercial or financial relationships that could be construed as a potential conflict of interest.

## Appendix: Interview Guide

(1) Could you start by telling me what kind of music you like?
(2) How important is music to you in your life?
(3) Is it something you listen to every day?
(4) Do you tend to focus on it most of the time or is it just in the background?
(5) How important are the lyrics in the music?
(6) Do you ever find yourself listening to music to try to influence your mood one way or another?
(7) What sort of effect would it have on you when you do that?
(8) Would you find it annoying to listen to something upbeat when you are feeling low?
(9) Have you ever listened to music that made you feel worse?
(10) Do you think its possible that sometimes music could make you feel worse? If so, what kind? When and how?
